# BI-RADS Reading of Non-Mass Lesions on DCE-MRI and Differential Diagnosis Performed by Radiomics and Deep Learning

**DOI:** 10.3389/fonc.2021.728224

**Published:** 2021-11-01

**Authors:** Jiejie Zhou, Yan-Lin Liu, Yang Zhang, Jeon-Hor Chen, Freddie J. Combs, Ritesh Parajuli, Rita S. Mehta, Huiru Liu, Zhongwei Chen, Youfan Zhao, Zhifang Pan, Meihao Wang, Risheng Yu, Min-Ying Su

**Affiliations:** ^1^ Department of Radiology, The Second Affiliated Hospital, Zhejiang University School of Medicine, Hangzhou, China; ^2^ Department of Radiology, The First Affiliated Hospital of Wenzhou Medical University, Wenzhou, China; ^3^ Department of Radiological Sciences, University of California, Irvine, Irvine, CA, United States; ^4^ Department of Radiation Oncology, Rutgers-Cancer Institute of New Jersey, Robert Wood Johnson Medical School, New Brunswick, NJ, United States; ^5^ Department of Radiology, E-DA Hospital and I-Shou University, Kaohsiung, Taiwan; ^6^ Department of Medicine, University of California, Irvine, Irvine, CA, United States; ^7^ Zhejiang Engineering Research Center of Intelligent Medicine, The First Affiliated Hospital of Wenzhou Medical University, Wenzhou, China; ^8^ Department of Medical Imaging and Radiological Sciences, Kaohsiung Medical University, Kaohsiung, Taiwan

**Keywords:** breast neoplasms, computer-assisted diagnosis, deep learning, machine learning, magnetic resonance imaging

## Abstract

**Background:**

A wide variety of benign and malignant processes can manifest as non-mass enhancement (NME) in breast MRI. Compared to mass lesions, there are no distinct features that can be used for differential diagnosis. The purpose is to use the BI-RADS descriptors and models developed using radiomics and deep learning to distinguish benign from malignant NME lesions.

**Materials and Methods:**

A total of 150 patients with 104 malignant and 46 benign NME were analyzed. Three radiologists performed reading for morphological distribution and internal enhancement using the 5th BI-RADS lexicon. For each case, the 3D tumor mask was generated using Fuzzy-C-Means segmentation. Three DCE parametric maps related to wash-in, maximum, and wash-out were generated, and PyRadiomics was applied to extract features. The radiomics model was built using five machine learning algorithms. ResNet50 was implemented using three parametric maps as input. Approximately 70% of earlier cases were used for training, and 30% of later cases were held out for testing.

**Results:**

The diagnostic BI-RADS in the original MRI report showed that 104/104 malignant and 36/46 benign lesions had a BI-RADS score of 4A–5. For category reading, the kappa coefficient was 0.83 for morphological distribution (excellent) and 0.52 for internal enhancement (moderate). Segmental and Regional distribution were the most prominent for the malignant group, and focal distribution for the benign group. Eight radiomics features were selected by support vector machine (SVM). Among the five machine learning algorithms, SVM yielded the highest accuracy of 80.4% in training and 77.5% in testing datasets. ResNet50 had a better diagnostic performance, 91.5% in training and 83.3% in testing datasets.

**Conclusion:**

Diagnosis of NME was challenging, and the BI-RADS scores and descriptors showed a substantial overlap. Radiomics and deep learning may provide a useful CAD tool to aid in diagnosis.

## Introduction

Breast MRI is an important modality for the detection and characterization of lesions. It has become a clinical examination routinely used with mammography and ultrasound for diagnosis of breast cancer (1, 2). Dynamic contrast-enhancement MRI (DCE-MRI) is a well-established imaging method to evaluate the vascular properties, which can be used for distinguishing benign from malignant lesions (3, 4). In cases that mammography and ultrasound show equivocal results, MRI can provide important information for guiding the next procedure, such as biopsy or short-term follow-up (2). According to Breast Imaging Reporting and Data System (BI-RADS) (5), breast lesions on MRI are divided into three categories, i.e., focus, mass, and non-mass enhancement (NME). While mass lesions can be easily detected by all imaging modalities and diagnosed with a high accuracy, the diagnosis of NME lesions are more challenging (6). On mammography, since there is no mass effect, the diagnosis has to rely on the distribution of tissue density and/or micro-calcifications. On MRI, NME may show strong enhancements and can be reliably detected as suspicious when it is asymmetric between bilateral breasts. However, to further designate the detected NME as likely to be malignant or benign is more difficult, and machine learning-based computer-aided diagnosis (CAD) may provide a feasible tool (6).

Breast MRI abnormalities are usually interpreted by radiologists based on the evaluation of morphological features and DCE kinetic patterns with the assistance of DCE-specific display software, which is subjective and varies with radiologists’ experience (7). For NME, the fifth edition of BI-RADS has further revised the categories for morphological distribution and internal enhancement pattern. However, while some descriptors were strongly associated with malignancy, there was a substantial overlap between malignant and benign lesions, and it is difficult to make an accurate diagnosis (8–12). This problem was well recognized, and several CAD methods have been developed, specifically considering the different imaging features for mass and NME, respectively (13, 14).

In recent years, radiomics and machine learning have been extensively applied in the medical field. Radiomics analysis can extract many features from images and convert them into quantifiable data. Subsequently, machine learning algorithms can be applied to select important features to build models, e.g., for making a differential diagnosis (15–17). Deep learning using convolutional neural network (CNN) is also emerging rapidly, and it has been shown as a feasible method for diagnosis without using pre-defined feature extraction algorithms. ResNet is a commonly selected deep learning network for analysis of MR images, and it has been shown to be capable of diagnosing mass lesions with a high accuracy (16, 17), but studies dedicated to the diagnosis of NME were rarely reported.

The objective of this study was to implement radiomics and deep learning using ResNet50 to build diagnostic models for distinguishing malignant from benign NME on MRI. Three radiologists performed reading to give morphological distribution and internal enhancement pattern based on the BI-RADS lexicon. The diagnostic implication of the BI-RADS descriptors and the performance of radiomics and deep learning models were reported.

## Materials and Methods

### Patients

This was a retrospective study approved by the Institutional Review Board, and informed consent was waived. The NME was a lesion showing enhancement in an area that did not meet the definition for a mass (i.e., space-occupying lesions with distinct shapes and margins). The cases were selected from consecutive patients receiving breast MRI for diagnosis from January 2017 to December 2019, as centrally reviewed and determined by a radiologist (JZ) with 12 years of experience. The inclusion criteria were patients presenting non-mass enhancement lesions, who had histopathologically confirmed diagnosis *via* biopsy or surgery. All benign lesions in this study showed MR contrast enhancements and were confirmed histologically, not determined by follow-up. A total of 175 NME were identified. The exclusion criteria were patients receiving any prior treatment (*N* = 14), or with poor image quality and severe motion artifacts (*N* = 11). Finally, a total of 150 patients, including 104 with malignant cancers (mean age 49 ± 11, range 22 to 71 years old), and 46 patients with benign lesions (mean age 45 ± 12, range 23 to 80 years old), were included in the analysis. The mean 1-D tumor size (the maximum tumor size measured on the DCE-MRI) was 4.3 ± 2.0 cm (range 0.7 to 10.2 cm) in the malignant group and 2.3 ± 1.9 cm (range 0.5 to 7.3 cm) in the benign group. The histopathological types and the diagnostic BI-RADS score reported in the MRI report are listed in **Table 1**.

**Table 1:** The pathological types and diagnostic BI-RADS scores in malignant and benign groups.

	Groups	Case Number (%)
**Pathological Type**	**Malignant**	**Total *N* = 104**
	Invasive Ductal Cancer^†^	56 (53.8%)
	Ductal Carcinoma *In Situ* ^‡^	44 (42.3%)
	Other Invasive Cancer	4 (3.8%)
	**Benign**	**Total *N* = 46**
	Adenosis (Fibrocystic Changes)	28 (60.9%)
	Inflammation	7 (15.2%)
	Adenosis + Intraductal Papilloma	5 (10.9%)
	Adenosis + Fibroadenoma	3 (6.5%)
	Fibroadenoma	2 (4.3%)
	Adenosis + Inflammation	1 (2.2%)
**Diagnostic BI-RADS Score**	**Malignant**	**Total *N* = 104**
	BI-RADS 4A	8 (7.7%)
	BI-RADS 4B	17 (16.3%)
	BI-RADS 4C	26 (25.0%)
	BI-RADS 5	53 (51.0%)
	**Benign**	**Total *N* = 46**
	BI-RADS 3	10 (21.7%)
	BI-RADS 4A	14 (30.4%)
	BI-RADS 4B	16 (34.8%)
	BI-RADS 4C	4 (8.7%)
	BI-RADS 5	2 (4.3%)

^†^Main pathology is IDC, may have presence of DCIS or invasive lobular cancer.

^‡^Main pathology is DCIS, may contain micro invasion of IDC.

### MRI Protocol

Breast MRI was performed using a GE 3.0T system with an eight-channel breast coil. DCE-MRI was acquired using the three-dimensional volume imaging for breast assessment (VIBRANT) sequence in axial view to cover both breasts, with TR = 5 ms; TE = 2 ms; FA = 10°; slice thickness = 1.2 mm without gap; FOV = 34 × 34 cm^2^; and matrix size = 416 × 416. The DCE-MRI series consisted of six frames: one pre-contrast (F1) and five post-contrast (F2–F6). The contrast agent, gadopentetate dimeglumine (Magnevist; Bayer Schering Pharma), at a dosage of 0.1 mmol/kg, was intravenously injected after the pre-contrast frame was acquired, followed by 20 ml saline flush. The injection rate was 2.5 ml/s using a power injector, so the injection of the contrast medium and saline could be completed in 20 s. The acquisition time for each DCE frame was 1 min 32 s. The k-space was sampled using the rectilinear trajectory, with the central line acquired in the middle at 46 s.

### BI-RADS Reading for NME Descriptors

**Figure 1:**
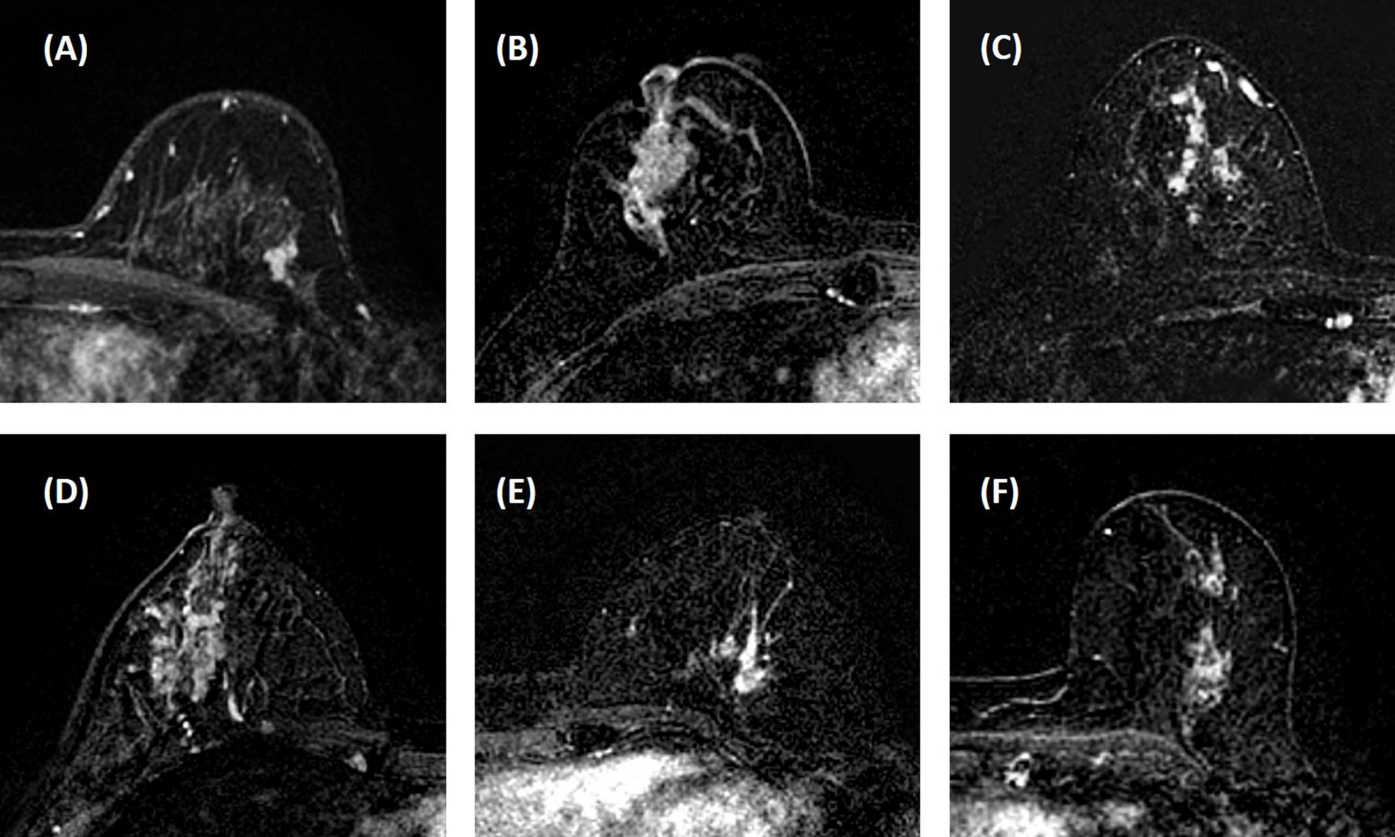
Case examples of morphological distribution and internal enhancement pattern evaluated based on the 5th BI-RADS lexicon. **(A)** Forty-seven-year-old diagnosed with adenosis, showing focal distribution and homogeneous enhancement. **(B)** Forty-one-year-old diagnosed with ductal carcinoma *in situ* (DCIS), showing segmental distribution and heterogeneous enhancement. **(C)** Forty-three-year-old diagnosed with invasive ductal cancer (IDC), showing segmental distribution and clustered ring enhancement. **(D)** Thirty-six-year-old diagnosed with inflammation, showing regional distribution and heterogeneous enhancement. **(E)** Fifty-one-year-old diagnosed with IDC, showing regional distribution and clumped enhancement. **(F)** Fifty-two-year-old diagnosed with DCIS, showing multiple distributions and clumped enhancement. In these six cases, all three readers give consistent BI-RADS category results.

The morphological distribution and internal enhancement pattern were read independently by three radiologists (JZ, HL, and ZC, with 12, 7, and 6 years of experience interpreting breast MRI), according to the 5th BI-RADS lexicon for NME lesions. They were blind to the histopathological results, other imaging findings (mammography and/or ultrasound, if any), and clinical diagnosis. The BI-RADS category for the morphological distribution included Focal, Linear, Segmental, Regional, Multiple, and Diffuse; and that for the internal enhancement pattern included Homogeneous, Heterogeneous, Clumped, and Clustered ring. Reference images showing each of the morphological distribution and internal enhancement pattern, following examples in Lunkiewicz et al. (12), were prepared and shown to the readers for training, before they were confident to start the reading. Six case examples illustrating these descriptors are shown in **Figure 1**.

### Tumor Segmentation

The radiologist who centrally reviewed and determined NME lesions (JZ) performed the segmentation. For each case, the location and the slice range that contained the tumor were decided, and then the tumor ROI was automatically segmented on contrast-enhanced maps by using the fuzzy-C-means (FCM) clustering algorithm with 3D connected-component labeling, as described previously (13, 17, 18). In 45 randomly selected cases, the lesion location and range information were provided to another radiologist (YZ, 6 years of experience) to perform segmentation, and the obtained radiomics features from these two sets of ROI’s were compared to test the feature reproducibility by using the intra-class coefficient (ICC).

### Radiomics Analysis

**Figure 2:**
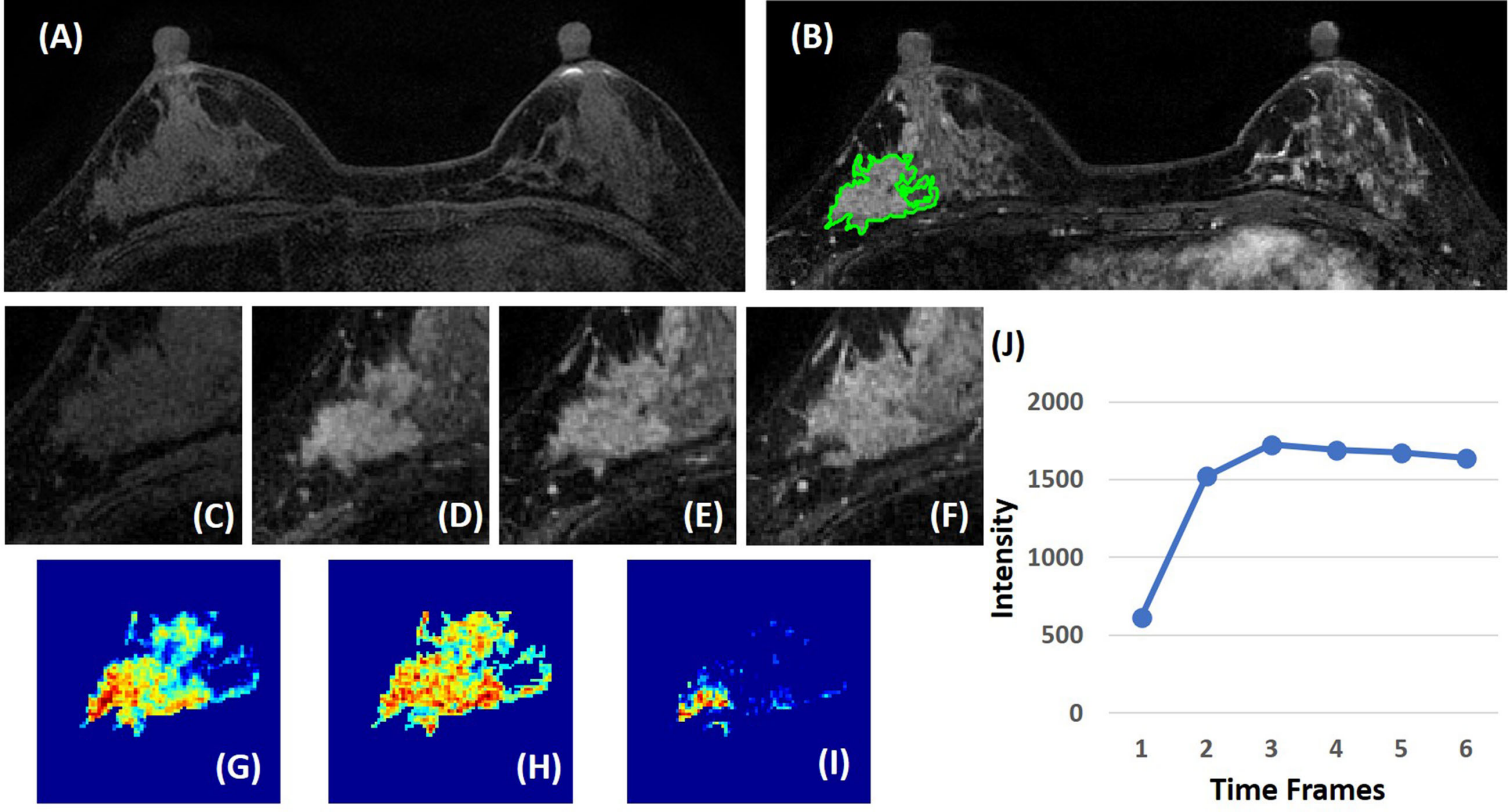
A 41-year-old patient with ductal carcinoma *in situ* (DCIS). **(A)** F1 pre-contrast image. **(B)** F2 post-contrast image. **(C–I)**: The zoom-in smallest bounding box containing the tumor. **(C)** F1 pre-contrast, **(D)** F2 post-contrast, **(E)** F3 post-contrast, **(F)** The last F6 post-contrast image, showing a comparable enhancement as in F3. **(G)** The wash-in signal enhancement map F2–F1. **(H)** The maximum F3–F1 signal enhancement map. **(I)** The wash-out F6–F3 map. **(J)** The DCE time course shows a plateau pattern, after reaching the maximum in F3.

**Figure 3:**
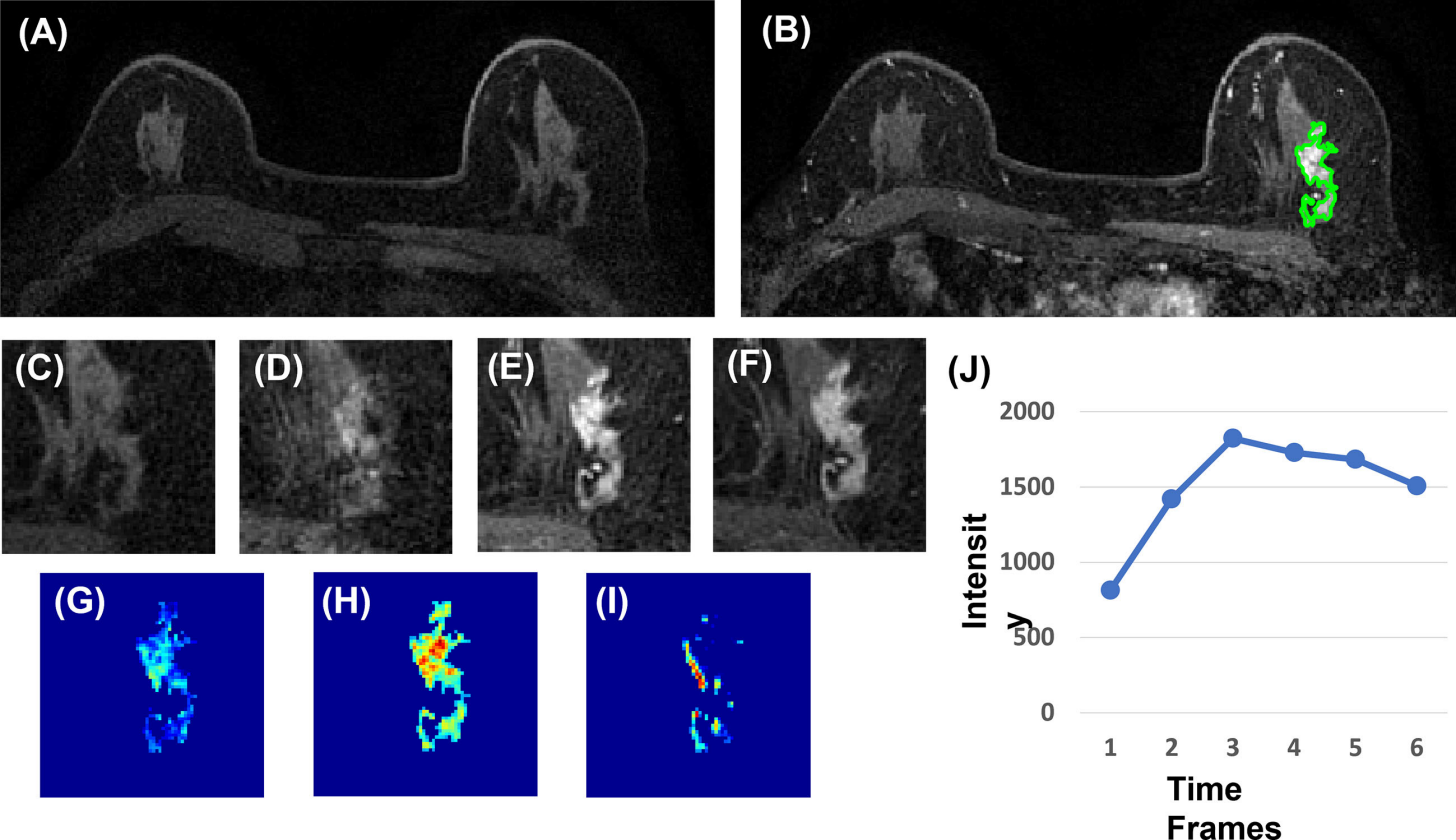
A 63-year-old patient with invasive ductal cancer (IDC). **(A)** F1 pre-contrast image. **(B)** F2 post-contrast image. **(C)** F1 pre-contrast. **(D)** F2 post-contrast. **(E)** F3 post-contrast. **(F)** The last F6 post-contrast image, showing wash-out DCE pattern with decreased intensity after reaching maximum in F3. **(G)** The wash-in signal enhancement map F2–F1. **(H)** The maximum F3–F1 signal enhancement map. **(I)** The wash-out F6–F3 map. **(J)** The DCE time course shows a typical wash-out pattern, reaching maximum in F3, followed by decreased intensity from F4 to F6.

**Figure 4:**
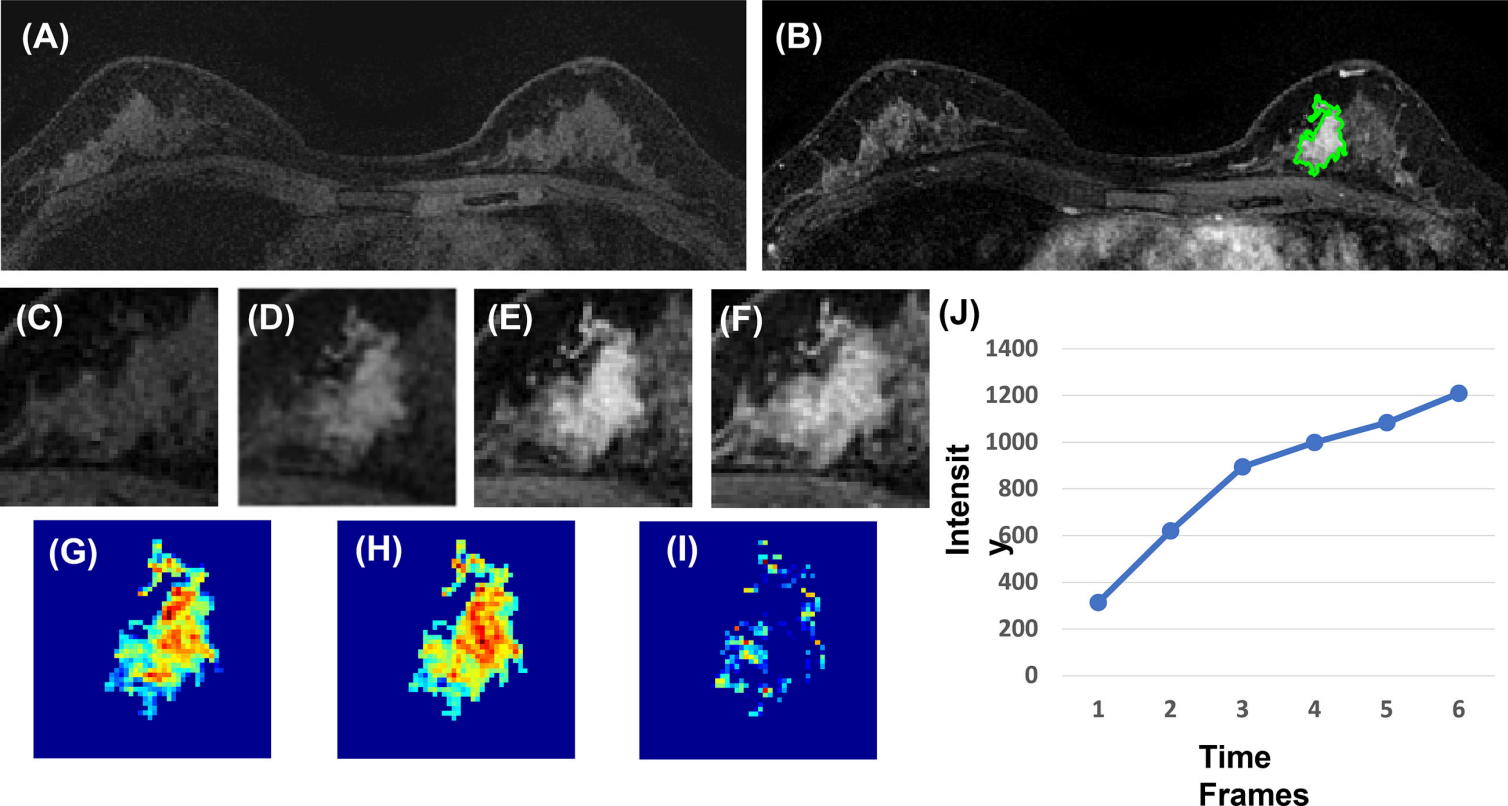
A 40-year-old patient with benign adenosis. **(A)** F1 pre-contrast image. **(B)** The F2 post-contrast image. **(C)** F1 pre-contrast. **(D)** F2 post-contrast. **(E)** F3 post-contrast. **(F)** The last F6 post-contrast image, showing persistent enhancement with increased intensity over time. **(G)** The wash-in signal enhancement map F2–F1. **(H)** The F3–F1 signal enhancement map. **(I)** The wash-out F6–F3 map. **(J)** The DCE time course shows a persistent enhancement pattern from F1 to F6.

**Figure 5:**
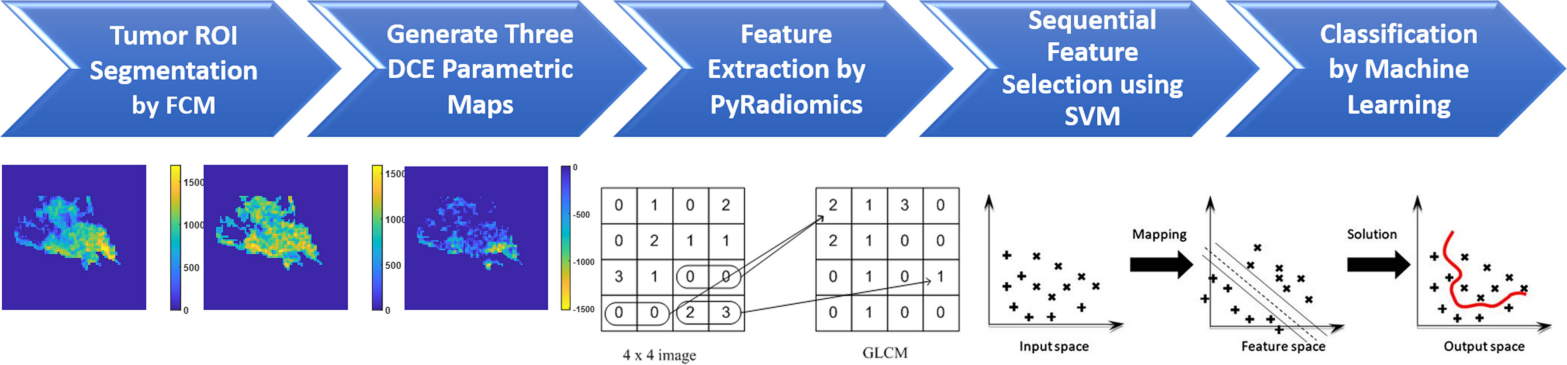
The flowchart of the radiomics analysis procedures. The tumor is first segmented on F2–F1 subtraction image using the FCM algorithm, and then the tumor ROI is mapped to three generated DCE parametric maps. On each map, 107 parameters are extracted using PyRadiomics. They are used for feature selection by SVM, and then for building classification models using five different machine learning algorithms.

Three heuristic DCE parametric maps defined below were generated: Wash-in Signal Enhancement (SE) Map = [(F2 − F1)/F1]; Maximum SE Map = [(F3 − F1)/F1]; Wash-out Slope Map = [(F6 − F3)/F3], following the methods previously developed for diagnosis of mass lesions on breast MRI (17). These three maps could reveal important features associated with the wash-in and wash-out phases in the DCE period. Examples from two malignant lesions are shown in **Figures 2**, **3**, and one benign lesion is shown in **Figure 4**. The 3D tumor mask was interpolated to have isotropic voxel resolution. The radiomics analysis was performed using PyRadiomics, an open-source radiomics library written in Python (19). On each parametric map, 107 features were extracted, so there were a total of 321 parameters from three maps. Only 268 features showing ICC ≥ 0.8 were included in the final analysis. The analysis flowchart is shown in **Figure 5**.

The sequential feature selection was performed by constructing multiple support vector machine (SVM) classifiers (20). In this process, SVM with Gaussian kernel was used as the objective function to test the performance of a subset of features using 10-fold cross-validation. In the beginning, an empty candidate set was presented, and features were sequentially added. In each iteration, the training process was repeated 1,000 times to explore the robustness of each feature. After each iteration, the feature that led to the best performance was added to the candidate set. When the addition of features no longer met the criterion, the selection process stopped. We used 10^−6^ as the termination tolerance for the objective function value.

After features were selected by SVM, several machine learning algorithms were applied to build the classification models, including SVM with the Gaussian kernel, Decision Tree, K-Nearest Neighbor (KNN), Linear Discriminant Analysis (LDA), and Naïve Bayes (21–23). The diagnostic performance was evaluated using 10-fold cross-validation in the training dataset, and then the developed final model was applied to the held-out testing dataset. The cases were separated into training and testing sets according to the time of acquisition, earlier 70% of cases (72 malignant and 33 benign) for training and later 30% (32 malignant and 13 benign) held out for testing. Because the ratio between the number of malignant and benign lesions was approximately 2:1, the class weights of benign and malignant images were assigned as 2:1 for the feature selection process and classification models, so the results would not be biased by the higher number of malignant cases.

### Deep-Learning Analysis

Deep learning was performed using ResNet50 (24), following the procedures previously applied to diagnose mass lesions (17). Detailed methods about the network architecture and training/validation were reported there. Three DCE parametric maps were used as inputs. All pixels in one map were normalized to have mean = 0 and standard deviation = 1. The smallest bounding box encasing the lesion was resampled to 75 × 75 pixels as the input image. In deep learning, data augmentation was needed, which was done by using random affine transformations, including translation, scaling, and rotation. Each malignant slice was augmented 20 times and each benign slice was augmented 40 times to balance the cases. For training, the Adam optimizer was used and set to 0.001 (25). The batch size was set as 64. To prevent the overfitting, L2 regularization and early stopping were used. In the training dataset, 10-fold cross-validation was used to evaluate the performance, and then the developed final model was applied to the held-out testing dataset. Deep learning was performed using each slice as an independent input. After the slice-based analysis was completed, the highest probability among all slices of a lesion was assigned to that lesion to give a final lesion-based diagnosis using the probability threshold of ≥0.5 as malignant and <0.5 as benign.

### Statistical Analysis

Statistical analyses were performed using Matlab 2019b for Windows. For radiologists’ reading of morphological distribution and internal enhancement pattern, the kappa coefficient was used to assess the inter-observer agreement. Fisher’s exact test and odds ratio (OR) were used to compare the difference of each BI-RADS descriptor between malignant and benign groups. The radiomics and deep learning analysis yielded malignancy probability for each lesion in the training dataset, which was used to calculate sensitivity, specificity, and overall accuracy, and to generate the receiver operating characteristic (ROC) curve. Lastly, the trained model was applied to the held-out testing dataset to evaluate the performance.

## Results

### Pathological Types and Diagnostic BI-RADS Scores

The characteristics of the malignant and benign groups are shown in **Table 1**. The majority of malignant lesions were IDC and DCIS, and the most common benign pathology was adenosis (fibrocystic changes). In the MRI report, the final diagnostic BI-RADS scores for the malignant cases were all higher than 4, showing *N* = 8 (7.7%) BI-RADS 4A, *N* = 17 (16.3%) BI-RADS 4B, *N* = 26 (25.0%) BI-RADS 4C, and *N* = 53 (51%) BI-RADS 5. In the benign group, most of them also had scores higher than 4, showing *N* = 10 (21.7%) BI-RADS 3, *N* = 14 (30.4%) BI-RADS 4A, *N* = 16 (34.8%) BI-RADS 4B, *N* = 4 (8.7%) BI-RADS 4C, and *N* = 2 (4.3%) BI-RADS 5. If using the BI-RADS 4A as the cutoff, the sensitivity was 100%, but the specificity was only 21.7%.

### Morphology and Internal Enhancement BI-RADS Category

The BI-RADS category for morphology and internal enhancement was read by three radiologists independently to evaluate their agreement. For the morphological distribution, ĸ = 0.83, 95% confidence interval [0.81–0.84]; and for the internal enhancement pattern, κ = 0.52, 95% confidence interval [0.51–0.53]. The agreement was excellent for the distribution, but only moderate for the internal enhancement. When further reviewing the pathological types that were associated with inconsistent reading, there were no dominate types. The disagreement occurred in all types. The senior radiologist’s results are summarized in **Table 2**. Of the 104 malignant cases, Regional distribution was the most prominent (*N* = 45, 43.3%), followed by Segmental (*N* = 23, 22.1%). For enhancement, Heterogeneous (*N* = 45, 43.3%) and Clumped (*N* = 39, 37.5%) were two dominating types. Of the 46 benign lesions, Focal distribution (*N* = 28, 60.9%) and Heterogenous enhancement (*N* = 28, 60.9%) were the most prevalent types. These BI-RADS descriptors are illustrated in **Figure 1**. The positive predicting value (PPV) and odds ratio (OR) are calculated and listed in **Table 2**.

**Table 2:** BI-RADS category for morphology distribution and internal enhancement pattern in malignant and benign groups.

Features	Total Number	Malignant (*N* = 104)	Benign (*N* = 46)	PPV	*p*-value	Odds Ratio
**Morphology Distribution**						
Focal	45	17 (16.3%)	28 (60.9%)	37.8%	<0.001	0.13
Lineal	1	0 (0%)	1 (2.2%)	0%	0.308	0
Segmental	32	23 (22.1%)	9 (19.6%)	71.9%	0.833	1.17
Regional	51	45 (43.3%)	6 (13.0%)	88.2%	<0.001	5.08
Multiple Regions	19	17 (16.3%)	2 (4.3%)	89.5%	0.059	4.30
Diffuse	2	2 (1.9%)	0 (0%)	100%	1.000	Inf
**Internal Enhancement Pattern**						
Homogeneous	9	1 (1%)	8 (17.4%)	11.1%	<0.001	0.40
Heterogeneous	73	45 (43.3%)	28 (60.9%)	61.6%	0.052	1.32
Clumped	44	39 (37.5%)	5 (10.9%)	88.6%	<0.001	5.78
Clustered Ring	24	19 (18.3%)	5 (10.9%)	79.2%	0.340	2.07

PPV, positive predicting value.

The results combining the distribution and enhancement are shown in **Table 3**. Of the 104 malignant cases, the top three were Regional/Clumped (*N* = 28, 26.9%), Segmental/Heterogeneous (*N* = 17, 16.3%), and Focal/Heterogeneous (*N* = 16, 15.4%). Of the 46 benign cases, the top three were Focal/Heterogeneous (*N* = 19, 41.3%), Focal/Homogeneous (*N* = 7, 15.2%), and Segmental/Homogeneous (*N* = 6, 13.0%).

**Table 3:** Combined morphology distribution and internal enhancement pattern in malignant and benign groups.

Morphology/Internal Enhancement	Total Number	Malignant (*N* = 104)	Benign (*N* = 46)	*p*-value
Focal/Homogeneous	8	1 (1.0%)	7 (15.2%)	<0.001
Focal/Heterogeneous	35	16 (15.4%)	19 (41.3%)	<0.001
Linear/Homogeneous	1	0 (0%)	1 (2.2%)	0.308
Segmental/Homogeneous	6	0 (0%)	6 (13.0%)	<0.001
Segmental/Heterogeneous	18	17 (16.3%)	1 (2.2%)	0.012
Segmental/Clumped	7	5 (4.8%)	2 (4.3%)	1.000
Segmental/Clustered ring	1	1 (1.0%)	0 (0%)	1.000
Regional/Heterogeneous	11	10 (9.6%)	1 (2.6%)	0.172
Regional/Clumped	30	28 (26.9%)	2 (4.3%)	<0.001
Regional/Clustered ring	12	7 (6.7%)	5 (10.9%)	0.509
Multiple regional/Heterogeneous	2	1 (1.0%)	1 (2.2%)	0.523
Multiple regional/Clumped	6	6 (5.7%)	0 (0%)	0.181
Multiple regional/Clustered ring	11	10 (9.6%)	1 (2.2%)	0.168
Diffuse/Heterogeneous	1	1 (1.0%)	0 (0%)	1.000
Diffuse/Clustered ring	1	1 (1.0%)	0 (0%)	1.000

### Radiomics Analysis Using Various Machine Learning Algorithms

**Figure 6:**
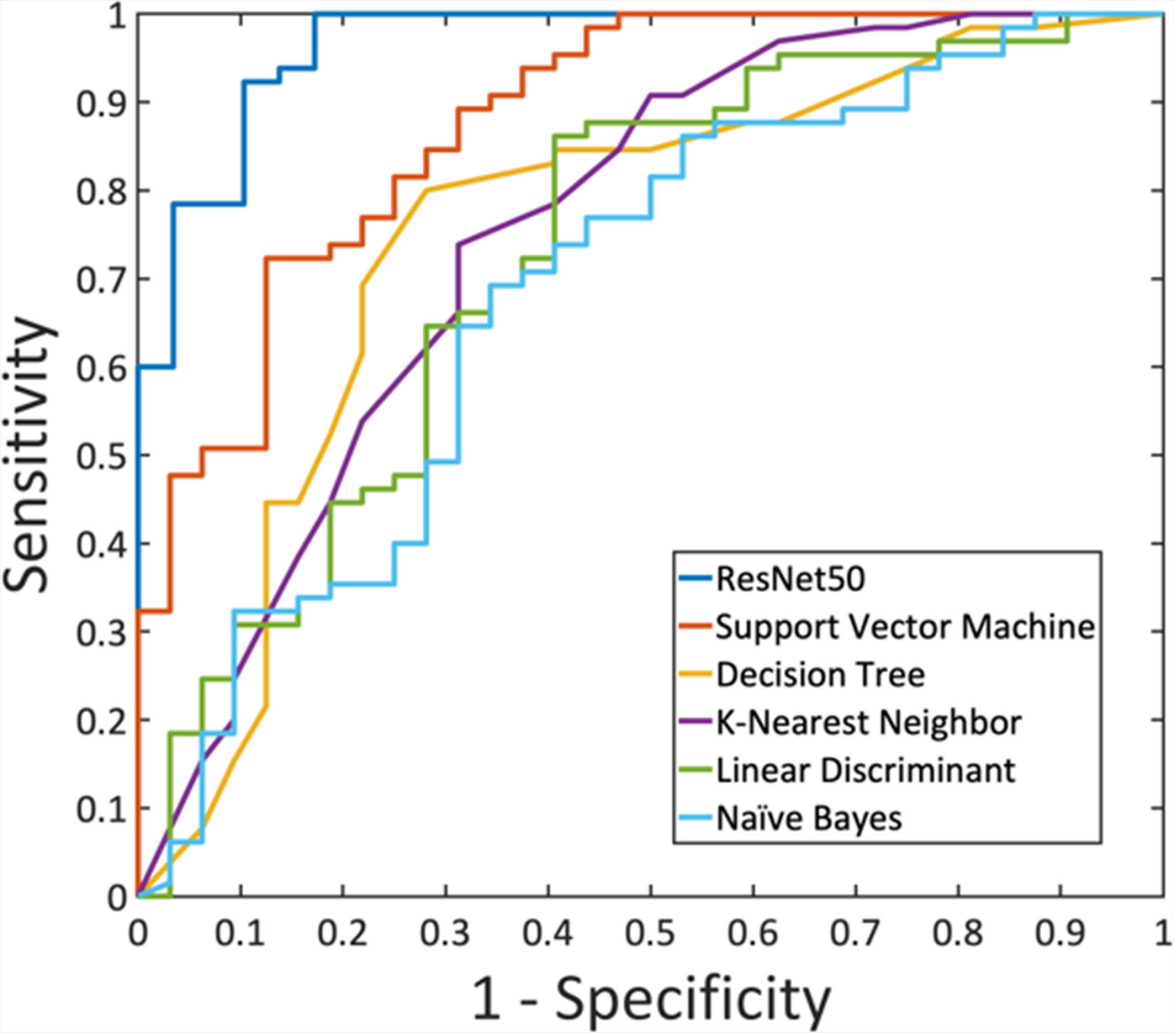
The ROC curves generated from the predicted per-lesion malignancy probability in the training dataset, by using ResNet50 and the five radiomics models built using: Support Vector Machine, Decision Tree, K-Nearest Neighbor, Linear Discriminant Analysis, and Naïve Bayes.

A total of eight radiomics features were selected by SVM, including (1) gray-level co-occurrence matrix (GLCM) autocorrelation from wash-out map, (2) gray-level size zone matrix (GLSZM) small area high gray level emphasis from wash-in map, (3) GLCM difference entropy from wash-out map, (4) GLCM autocorrelation from maximum SE map, (5) gray-level run length matrix (GLRLM) short run emphasis from maximum SE map, (6) gray-level dependence matrix (GLDM) high gray level emphasis from maximum SE amp, (7) GLDM dependence non-uniformity normalized from wash-in map, and (8) GLCM joint average from wash-in map. These features were used to build the model, and the results are summarized in **Table 4**. In the training dataset, the achieved accuracy was 80.4%, 77.3%, 75.3%, 75.3%, and 71.1% for SVM, Decision Tree, KNN, LDA, and Naïve Bayes, respectively. The ROC curves for all models are generated and shown in **Figure 6**. When these models were applied to the held-out testing dataset, the accuracy was 77.5%, 75.0%, 67.5%, 70.0%, and 62.5%, respectively.

**Table 4:** Diagnostic sensitivity, specificity, and accuracy using models built by ResNet50 deep learning and radiomics with five different machine learning algorithms.

	Training Dataset (10-fold cross-validation)				Testing dataset		
	Sensitivity	Specificity	Accuracy	AUC	Sensitivity	Specificity	Accuracy
**ResNet50**	95.4%	82.8%	91.5%	0.97	88.9%	66.7%	83.3%
**Radiomics**							
**SVM (Gaussian)**	98.5%	43.8%	80.4%	0.88	92.9%	41.7%	77.5%
Decision Tree (Coarse)	84.6%	62.5%	77.3%	0.75	85.7%	50.0%	75.0%
KNN (Cosine)	90.8%	43.8%	75.3%	0.75	71.4%	58.3%	67.5%
Linear Discriminant	84.6%	56.3%	75.3%	0.73	78.6%	50.0%	70.0%
Naïve Bayes (Gaussian)	83.1%	46.9%	71.1%	0.69	57.1%	75.0%	62.5%

### Deep Learning Analysis Using ResNet50

For each lesion, the smallest bounding box covering the tumor on all slices was generated, as illustrated in **Figures 2**–**4**. The three cropped parametric maps were used as input into the ResNet50 network. The ROC curve is shown in **Figure 6**. The per-lesion diagnostic results are summarized in **Table 4**. In the training dataset, sensitivity = 95.4%, specificity = 82.8%, and accuracy = 91.5% with an AUC of 0.97. When the developed model was applied to the held-out testing dataset, sensitivity = 88.9%, specificity = 66.7%, and accuracy = 83.3%. The performance of ResNet50 was better than all radiomics models.

## Discussion

The detection and diagnosis of NME have been known as a more challenging problem compared to mass lesions, which may be addressed by advanced machine learning methods (6). The common histopathology that may manifest as NME includes ductal carcinoma *in situ* (DCIS), invasive ductal cancer (IDC), invasive lobular cancer (ILC), benign adenosis, fibrocystic changes, and inflammation (12, 26). For NME, cancerous tissues were admixed with fibrotic tissues and there were no clear boundaries. Only enhanced benign lesions meeting the criterion of NME were included in this study, and all of them had a diagnostic BI-RADS score ≥ 3, with the majority of them (36/46 = 78.3%) having BI-RADS ≥ 4. All patients received biopsies and had histologically confirmed benign diagnoses. The results show that this is a difficult patient population for diagnosis, which is the objective of the study to explore the performance of advanced CAD using radiomics and deep learning.

The specific descriptors for NME lesions revised in the 5th BI-RADS lexicon were used. Our reading results show that regional and segmental were the two dominating morphology types. The two main enhancement categories were heterogeneous and clumped, but the readers could not reach a good agreement between them, only reaching a moderate kappa value of 0.52. NME was known to present heterogeneous enhancement. Whether it was clumped or not was related to the degree of heterogeneity, i.e., whether there was the presence of aggregated bright spots, and it was subjective. There was no clear graphical depiction similar to those illustrating morphological distribution for readers to follow (12). Besides, even with the precise classification of morphological distribution and enhancement patterns, they were not related to a clear distinction between malignant and benign lesions, as demonstrated in our results in **Tables 2**, **3**. For mass lesions, several prominent features, such as spiculation and rim enhancement, were highly suggesting malignancy, but none of the morphological or enhancement NME features had such a high PPV, and thus, more advanced CAD methods may be developed to help.

Motivated by the difficulty, we performed radiomics and deep learning to analyze this dataset and compared their diagnostic performance. The radiomics features were extracted using PyRadiomics, and eight features were selected by SVM for building models using five different machine learning algorithms. The results showed that SVM with Gaussian kernel had the highest accuracy compared to other algorithms, 80.4% in training, and 77.5% in testing datasets. With the feasibility of deep learning for the diagnosis of breast lesions on MRI (16, 17, 27), we applied ResNet50 to this NME dataset to investigate the performance. The accuracy was 91.5% in the training, and 83.3% in the testing datasets. Similar to reported studies in the literature (16, 17, 28, 29), in general, deep learning had a better diagnostic performance compared to radiomics models (**Figure 6**), presumably because of the high level of flexibility not limited by the pre-defined features extracted using certain computer algorithms.

Radiomics is based on the assumption that extracted imaging data are the product of mechanisms occurring at a genetic and molecular level linked to the genotypic and phenotypic characteristics of the tissue/tumor (30). On the other hand, deep learning uses convolutional neural networks to provide an efficient method in imaging processing, which can be applied to perform many clinical classification tasks (31–33). Due to distinctively different features, it is known that the diagnosis for mass and NME has to be done with separate computer-aided models (13, 14). Most machine learning breast MRI CAD studies, either done using radiomics or deep learning, did not separate them (15, 16). To our best knowledge, so far there has not been any machine learning-based CAD study dedicated to systematically compare the diagnosis of NME using radiomics and deep learning; therefore, no results could be compared to ours.

Regarding the specific reading results, the focal distribution and homogeneous enhancement were significant benign predictors, while regional, multiple, and clumped enhancement were significant malignant predictors. Diffuse distribution and clustered ring enhancement also had high PPV, but the case number was too small to reach a significant level. Several studies have compared the NME descriptors analyzed using the fifth edition of BI-RADS lexicon (8–12), and the reported results were pretty consistent. However, since the patient population was very different, the percentage of features and PPV could not be directly compared. When a screening population was analyzed, more benign cases would be included (9, 11, 12), but when a diagnostic population was analyzed as in our study, more malignant cases were found (8, 10). In these studies, and earlier studies using the previous BI-RADS lexicon, the clumped and clustered ring enhancements were reported to have a high PPV associated with malignancy. In our study, the clustered ring was seen in 19/104 (18.3%) malignant patients, but 5/46 (10.9%) benign patients also showed this feature. Interestingly, of these five patients, four patients had confirmed inflammation, which could be very difficult to diagnose. Particularly, inflammation tended to be larger and presented segmental, regional, and multiple distributions, as in the case shown in **Figure 1D**. Extensive fibrocystic changes were also likely to present as NME, mimicking malignancy in visual evaluation (34).

There were some limitations. First of all, the case number was small, especially for radiomics and deep learning analysis. Considering that NME was fewer than mass lesions, it was difficult to assemble a large NME dataset that showed strong enhancements and had pathologically confirmed diagnosis for benign cases. That might be the main reason for the rare report in the literature. The case number may be expanded by including women receiving MRI for screening purposes, and adding benign lesions determined by follow-up without biopsy. However, mixing diagnostic and screening patients would lead to another bias problem, and the results might greatly depend on the composition of the analyzed patient population. Second, three radiologists reviewed the entire dataset to give BI-RADS category for morphology and internal enhancement, but they did not attempt to give a final diagnostic score, or the malignant vs. benign diagnosis. Instead, we reported the BI-RADS scores given in the initial MRI report. In future prospective studies, radiologists’ comprehensive reading can be done, and their diagnostic results can be compared to those analyzed by the radiomics or deep learning models, e.g., the model developed in this work. Lastly, the training and testing datasets were from the same hospital acquired using the same MR scanner. As such, the developed models may not be applicable to datasets acquired using a different scanner, or datasets from a different institution acquired using a different protocol. In a recent study, we showed that transfer learning is needed to fine-tune the models developed for predicting the breast cancer molecular subtypes using one dataset for a different dataset acquired from a different hospital (35). An external independent testing dataset is needed in the future to evaluate the performance of the developed models for diagnosis of NME here, and further to investigate how the model needs to be fine-tuned.

In conclusion, we assembled a breast MRI NME dataset and had three radiologists perform reading for the morphological distribution and internal enhancement pattern based on the 5th BI-RADS lexicon. The results showed that there was a substantial overlap between the BI-RADS features of malignant and benign NME lesions. Radiomics and deep learning methods were implemented to investigate their potential to provide a machine-learning based CAD tool, and a high accuracy was achieved. The value of AI models in diagnostic radiology is well recognized, mostly in providing additional information for the radiologist, not mature yet for making a diagnostic recommendation. Given the difficulty in the diagnosis of NME compared to mass lesions by radiologist’s reading, these tools may have a clinical value, which is rarely reported. The diagnostic model for NME will further complement those developed for mass lesions to demonstrate the clinical feasibility of the AI-based machine learning and deep learning algorithms for making differential diagnosis for all types of lesions detected by breast MRI.

## Data Availability Statement

The datasets used and analyzed in this study will be made available by the corresponding author on a reasonable request.

## Ethics Statement

The studies involving human participants were reviewed and approved by The Second Affiliated Hospital, Zhejiang University School of Medicine. Written informed consent for participation was not required for this study in accordance with the national legislation and the institutional requirements.

## Author Contributions

Study concept and design: JZ, RY, and M-YS. Acquisition of data: JZ, HL, ZC, YZ, ZP, MW. Analysis of data: JZ, Y-LL, J-HC, FJC, RP, RSM, ZC, YZ, M-YS. Drafting of the manuscript: JZ, Y-LL, YZ, J-HC, FJC, RP, RSM, M-YS. Critical revision: MW and M-YS. Statistical analysis: Y-LL and YZ. Study supervision: MW, RY, and M-YS. All authors contributed to the article and approved the submitted version.

## Funding

This work was supported in part by Research Incubation Project of First Affiliated Hospital of Wenzhou Medical University (No. FHY2019085), Zhejiang Provincial Natural Science Foundation of China (LY21F020030), Medical Health Science and Technology Project of Zhejiang Province Health Commission (No. 2019326177), and NIH/NCI R01 CA127927, R21 CA208938, P30 CA062203, and the UC Irvine Comprehensive Cancer Center using UCI Anti-Cancer Challenge funds. The content is solely the responsibility of the authors and does not necessarily represent the official views of the National Institutes of Health or the Chao Family Comprehensive Cancer Center.

## Conflict of Interest

The authors declare that the research was conducted in the absence of any commercial or financial relationships that could be construed as a potential conflict of interest.

## Publisher’s Note

All claims expressed in this article are solely those of the authors and do not necessarily represent those of their affiliated organizations, or those of the publisher, the editors and the reviewers. Any product that may be evaluated in this article, or claim that may be made by its manufacturer, is not guaranteed or endorsed by the publisher.
